# Digital ischemic events related to gemcitabine: Report of two cases and a systematic review

**DOI:** 10.2478/v10019-010-0020-1

**Published:** 2010-05-03

**Authors:** Cvetka Grasic Kuhar, Tanja Mesti, Branko Zakotnik

**Affiliations:** Department of Medical Oncology, Institute of Oncology Ljubljana, Ljubljana, Slovenia

**Keywords:** chemotherapy, gemcitabine vascular toxicity, digital ischemic events

## Abstract

**Background:**

Gemcitabine is a potent cytotoxic agent used in the treatment of many solid tumours, sarcomas and lymphomas. Vascular toxicity and thrombotic events related to gemcitabine seem to be underreported.

**Case report:**

We report two cases of gemcitabine related digital ischemic events.

*Case 1.* A 65-year-old man was given the first-line treatment with gemcitabine for the advanced adenocarcinoma of pancreas. After four weekly doses of gemcitabine (total dose 4000 mg/m^2^) he presented with Raynaud’s like phenomenon and ischemic fingertips necrosis in five digits of both hands. Symptoms resolved in all but one digit after stopping chemotherapy and treatment with iloprost trometamol infusion.

*Case 2.* A 77-year-old man, ex-smoker, was administered a combination of gemcitabine and cisplatin as the first-line treatment for the locally advanced bladder cancer. After 4 cycles of the treatment (total dose of gemcitabine 4000 mg/m^2^) the patient suffered digital ischemia and necrosis on two digits of a right leg. Arteriography revealed preexisting peripheral arterial occlusive disease (PAOD) of both legs with very good peripheral collateral circulation and absent microcirculation of affected two digits. The gemcitabine treatment was stopped and the patient was treated with iloprost trometamol infusion and percutaneous transluminal angioplasty with dilatation of the right superficial femoral artery. Digital changes resolved without consequences. Severe thrombocytosis (platelet count 1211 × 10^9^/L) might have also contributed to the ischemic digital event in the second case.

**Conclusions:**

Digital ischemic events associated with gemcitabine chemotherapy seem to be more common in patients with tobacco-associated cancers, especially when used in combination with platinum salt. The treatment with gemcitabine in patients with evolving Raynaud’s phenomenon and/or preexisting PAOD should be done with caution.

## Introduction

Gemcitabine is an important contemporary chemotherapeutic agent for the treatment of both solid tumours and lymphomas.^1^ As a nucleoside analog structurally related to cytosine arabinoside, it interferes with the synthetic pathways of the tumour cell predominantly in the S phase of the cell cycle. It is a potent inhibitor of DNA synthesis and repair.^2^

First approved in 1996 for the treatment of unresectable pancreatic cancer, today gemcitabine is most commonly used in the therapy of pancreatic, ovarian, breast, non-small cell lung and bladder cancer, some sarcomas, cutaneous and peripheral T-cell lymphomas as well as in relapsed Hodgkin’s lymphoma.^1^ The use of this drug enables progress in the routine management of cancer patients.^3^,^4^

Gemcitabine is a drug with a favourable toxicity profile with myelosuppression, a flu-like syndrome, skin rash and radiation recall dermatitis being the most common side effects.^2^

As indications for its use in oncology have been expanding, some reports showed the possibility of its vascular side effects. Among them thrombotic microangiopathy^5^, venous thrombembolism, acute arterial events (digital ischemia and necrosis, vasculitis), systemic capillary leak syndrome and reversible posterior leukoencephalopathy are reported.^1^

Digital ischemic events are rare in cancer patients. They are most frequently related to vascular disease due to hypercholesterolemia, arterial hypertension, diabetes or exposure to tobacco. They may also occur as a complication of connective tissue diseases, i.e. vasculitis with digital ischemic events.

In the present article we report two cases of digital ischemic events during the therapy with gemcitabine alone and in combination with cisplatin and review data in the literature regarding this rare side effect.

### Case 1

A 65-year-old male was presented in March 2009 with primary metastatic adenocarcinoma of pancreas (metastases in retroperitoneal and cervical nodes). He had a history of nephrolythiasis ten years ago and 5 months lasting history of hydronephrosis of the left kidney of grade III, caused by enlarged retroperitoneal lymph nodes. At presentation he was overweight with grade I renal impairment (creatinine 129 μmol/L). He complained of pain in the upper abdomen. Tumour marker CA 19-9 was elevated (> 12 000 IU); platelet count was 376 × 10^9^/L.

In March 16 2009 he started the treatment with gemcitabine monotherapy in a weekly dose 1000 mg/m^2^. After 3 weekly doses he had less pain in the abdomen and tumour marker CA 19-9 halved (6163 IU). Platelet count was elevated (570 × 10^9^/L). He complained of painful swelling in three digits of right hand and acrocyanosis. After the 4^th^ dose of gemcitabine pain in digits increased and the patient was admitted to the local hospital. Raynaud’s syndrome was suspected. Criteria for paraneoplastic microthrombosis, which was suspected, were not met. At the beginning the patient was treated with acetylsalicylic acid. No improvement was recorded. On the control visit in our institution (May 05 2009) the patient complained of severe pain in five digits of both hands. The examination showed dry fingertips necrosis. Radial and ulnar pulses were normal. The Doppler ultrasound of both arms showed normal macrocirculation. Digital plethismography showed an absent signal on digits I, II and IV of the right and digits III and V of the left hand. Gemcitabine induced vasculitis causing ischemia was suspected and gemcitabine treatment was stopped. The patient was treated with the prolonged infusion of a prostacycline analogue iloprost trometamol (20 mg/day for three weeks) and analgesic therapy with NSARD and opioids. Digital changes in all but one of affected digits resolved at the next visit in June 24 2009. Ischemic changes of distal phalange of digit V of the left hand required the amputation. The patient died in August 2009 due to the progressive disease.

### Case 2

**FIGURE 1.**
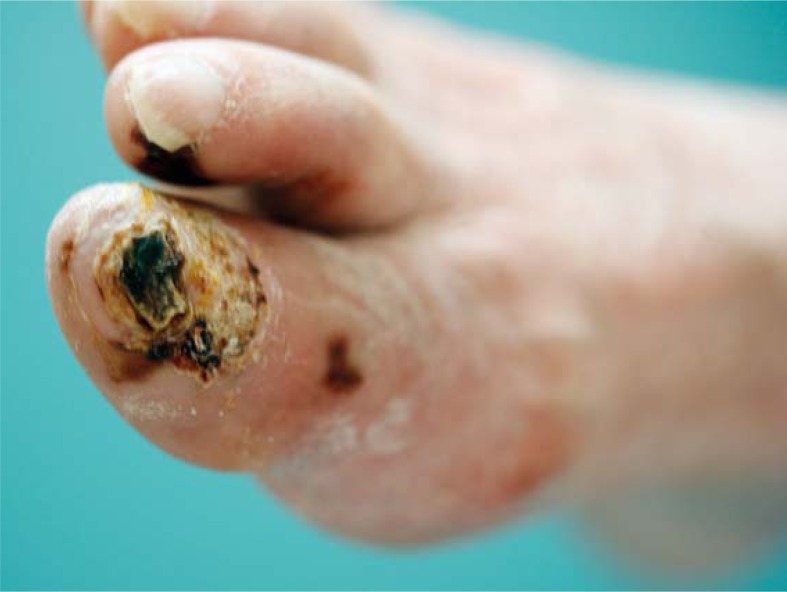
Ischemic necrosis on digits I and II of right foot after 4 cycles of chemotherapy with cisplatin and gemcitabine (Case 2).

**FIGURE 2.**
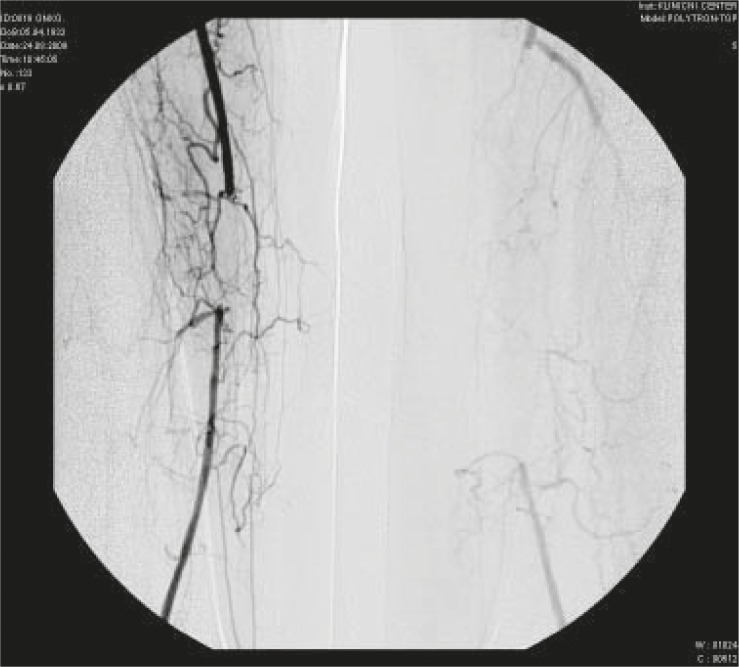
Pelvic arteriography showing occlusion of right superficial femoralis artery (AFS) in the length of 5 cm and of left AFS in length of 18 cm (Case 2).

**FIGURE 3.**
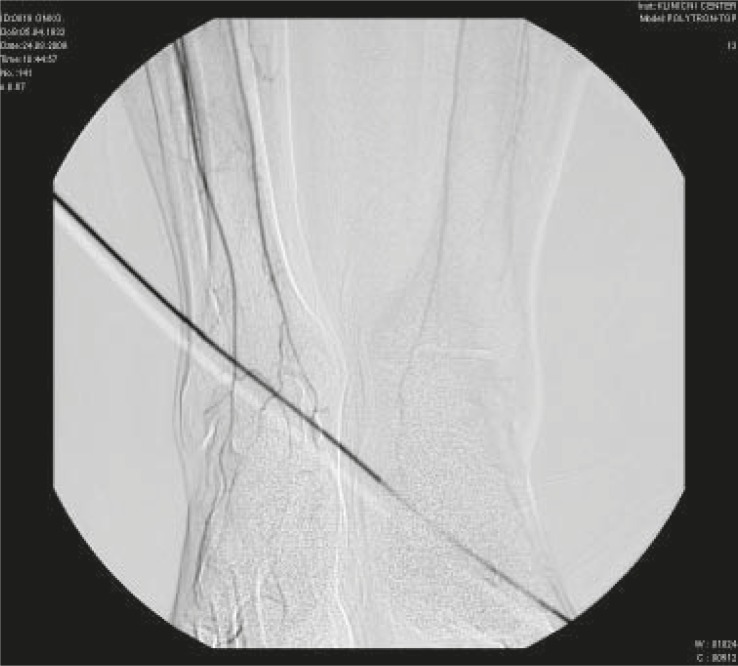
Arteriography showing impaired circulation of distal part of both legs in Case 2.

A 77- year-old male presented in May 06 2009 with a diagnosis of locally advanced bladder cancer (T4aN2M0). He was heavy a smoker in the past and had a history of gastric perforation due to peptic ulcer ten years ago. In February 2009 he was temporary on amiodarone medication due to paroxysm of atrial fibrillation. Otherwise he was in good physical condition. In April 2009 he underwent an attempt of radical cistoprostatectomy. Due to the local extension of the tumour only Bricker neovesica and biopsy of pelvic lymphnodes was performed. In May 2009 he was presented for induction chemotherapy and definitive radiotherapy afterwards. From May to August 2009 he received four cycles of chemotherapy with cisplatin and gemcitabine (cisplatin 75 mg/m^2^ on day 1 and gemcitabine 1000 mg/m^2^ on days 1 every three weeks). None of the planned gemcitabine doses on day 8 was applied due to the infection. In August 8 2009 the patient presented with painful black spots on digits I and II of the right foot and subluxation of the thumbnail of the same foot. He underwent the ablation of the thumbnail. Ischemic changes in the thumb were suspected. In August 18 2009 the patient presented with the progressive painful fingertip necrosis on fingertips I and II of the right foot (Figure 1). The elevated platelet count was recorded (1211×10^9^/L). He was sent to an angiologist. Doppler ultrasound showed severe peripheral arterial occlusive disease (PAOD) of both legs. Pelvic arteriography showed the occlusion of right superficial femoral artery (AFS) in the length of 5 cm and of left AFS in length of 18 cm, with very good collateral circulation on both sides and good transition of the both poplitheal arteries (Figure 2 and 3). The patient was treated with prolonged infusion of a prostacycline analogue - iloprost trometamol (20 mg/day for 7 days). A successful percutaneous transluminal angioplasty with dilatation of the right AFS was performed in September 1 2009. After this procedure the foot macrocirculation improved (warm skin of the right foot). Thereafter temporary wet necrosis and wound infection on digit II occurred, which healed until November 2 2009. The amputation of the affected digits was not required. Acetylsalicylic acid 100 mg/d was prescribed.

## Discussion

Digital ischemic events in cancer patients are rare. However, in cancer patients with a history of heavy smoking, dislypidemia, arterial hypertension or diabetes, PAOD may already be presented at cancer diagnosis and may lead to ischemic events. In systemic sclerosis the impairment of microcirculation due to vasculitis and/or vasospasm can also cause a digital arterial obstruction.^6^ Some anticancer agents (cyclophosphamide, methotrexate, 5-fluorouracil, vincristine, prednisone, doxorubicin, tamoxifen, cisplatin-gemcitabine combination) are implicated in thrombosis and thrombembolic events.^7^ Therefore, in a patient with predisposing factors for the impaired microcirculation these anticancer agents may attribute to digital ischemic events.^6^

Among cases in the literature, Barcelo *et al.*^7^ reported four cases of digital ischemic changes related to combination chemotherapy with gemcitabine plus cisplatin in patients treated for non-small cell lung cancer. In two of four cases a previously asymptomatic organic vascular lesion was aggravated while on chemotherapy with cisplatin and gemcitabine. Antiplatelet agent triflusal and vasoactive agent buflomedil were successful in resolving pain. One patient needed the additional leg amputation and two others thrombectomy.

Similarly, Venat-Bouvet *et al.*^8^ described a patient with the acute onset of bilateral PAOD with necrosis of fingerpads which presented after the treatment with gemcitabine and platinum salt as a first line treatment for urothelial carcinoma of the bladder. The patient had a favourable outcome after chemotherapy withdrawal and infusion of iloprost trometamol, as in our Case 2. In our Case 2 cumulative dose of gemcitabine was only 4000 mg/m^2^, in contrast to others (10000 mg/m^2^, 14390 mg).^8^,^9^

Blaise *et al.*^9^ reported two cases of digital ischemia. First was a female treated with gemcitabine as the second line for lymph-node metastatic squamous cell carcinoma of unknown origin. The second patient was treated for bladder carcinoma with gemcitabine and carboplatine. Both resolved after the interruption of gemcitabine and additional medical treatment.

Another case attributed to vascular toxicity of gemcitabine is reported by Holstein *et al.*^10^ treated with platinum plus gemcitabine for advanced urothelial carcinoma. After the second cycle of chemotherapy the patient presented with digital ischemia. Digital amputation was avoided by sympathicolysis by bilateral blockade of brachial plexus and application of iloprost, heparin, corticosteroids and acetylsalicylic acid.

Patients with scleroderma are at high risk for developing digital infarction because of their underlying vascular disease and associated Raynaud’s phenomenon. Clowse *et al.*^11^ presented a patient with scleroderma who developed multiple ischemic digits after receiving combination chemotherapy of carboplatin plus gemcitabine for lung cancer. D’Allesandro *et al.*^12^ reported a case with Raynaud type phenomenon, intermittent fever, digital necrosis and a fingertip gangrene after receiving two applications of gemcitabine for bladder cancer. The authors suggested caution in using such chemotherapy in subjects with autoimmune disorders (scleroderma, positive HEP-2 and cryoglobulin).^11^,^13^

Digital ischemic changes resolved in most cases after stopping the application of chemotherapy and the treatment with prostacyclin antagonists (iloprost or buflomedil). A more sophisticated treatment with sympathicolysis by bilateral thoracic block was reported to be efficient in digital ischemic event in scleroderma patient, where gangrenous ulcers occurred due to vaso-occlusive disease, which is a combination of occlusive vasculitis and symphatically-mediated vasospasm.^6^ Sympathectomy and iloprost infusion were reported to be successful in the treatment of severe acral ischemia and necrotic lesions of several fingertips after receiving palliative chemotherapy with gemcitabine for inoperable squamous cell carcinoma of the tonsil.^14^

In our Case 1 the first signs of the impaired digital circulation (swollen and painful cold blue fingers) occurred after three weekly doses of gemcitabine therapy and worsened after the next weekly application of the same drug, therefore the causal relationship between gemcitabine and digital arterial ischemia seems very probable. The patient had no history or symptoms of preexsisting PAOD or connective tissue disease. The Raynaud’s phenomenon can be a paraneoplastic manifestation in disseminated pancreatic cancer, but would already be present at presentation of disease and would probably improve with the effective anticancer therapy. The occurrence of digital ischemia was probably related to vascular toxicity of gemcitabine, as indicated by resolving microcirculation in 4 of 5 affected digits after the discontinuation of gemcitabine and the intervention with vasodilating agent iloprost.

In Case 2 after the cumulative dose of gemcitabine of 4000 mg/m^2^ and cisplatin 300 mg/m^2^ arterial ischemic necrosis on two digits of the right foot occurred. After a detailed investigation of the patient, severe preexisting PAOD of both legs with the very good collateral circulation was diagnosed. Gemcitabine may also be capable to cause endothelial damage and thrombocytosis.^1^,^2^ The latter could also attribute to impaired microcirculation in our case (platelet count at 3^rd^ cycle of chemotherapy 1211 × 10^9^/L). At that time the patient was on fractionated heparin in prophylactic dosing. Instead of heparin, the antiagregation therapy with acetylsalicylic acid would be more appropriate in preventing digital arterial thrombosis. After the dilatation of the occluded segment of the main leg arterial vessel and vasodilatation effect of iloprost infusion both macro and microcirculation improved, respectively. Digital ischemic necroses resolved without durable consequences.

In both cases painful cold trophic skin changes were clinically suspicious of compromised arterial microcirculation, confirmed by absent plethysmographic signals. In Case 1 Doppler ultrasound showed good macrocirculation, which was severely impaired in Case 2, as already suggestive of PAOD by patient’s long smoking history. Clinically absent pedal pulses and angiologic examination with arteriography could place angioplastic intervention before the initiation of chemotherapy with gemcitabine and cisplatin. A noninvasive evaluation of the leg arterial perfusion could be done by MRI enhanced angiography^15^ and an invasive angiography being applied only in cases where an invasive procedure is indicated. The antiagregation therapy (acetylsalicylic acid 100 mg a day) in case of severe reactive thrombocytosis in cancer patients as well as in patients with cardiovascular factors is indicated and could prevent the development of digital ischemia in Case 2. Discontinuation of cisplatin plus gemcitabine and the intervention with iloprost prometanol have been proven helpful in resolving microcirculation in both cases as documented by control plethysmography. Additionally calcium channel blockers and other vasodilatators were shown beneficial in resolving digital ischemia.^6^,^7^,^11^

## Conclusions

Digital ischemic events associated with gemcitabine chemotherapy seem to be more common than previously thought, especially when used in combination with platinum salts and in patients with tobacco-associated cancers. In patients with known risk factors for PAOD, like dislypidemia, arterial hypertension, diabetes or tobacco smoking, and a history of intermittent claudication thorough the examination of peripheral pulses should be performed before the initiation of gemcitabine or platinum-gemcitabine doublet. In addition, if painful trophic digital changes develop while on therapy with gemcitabine, medical oncologists should refer the patient to an angiologist for the assessment of impaired micro or macrocirculation. In case of diagnosis of ischemic vascular event, discontinuation of gemcitabine and immediately therapy with prostacycline analogues should be initiated. The early diagnosis and the appropriate intervention improve not only the outcome of the ischemic vascular event but also the quality of life of the patient.
